# Echolocating bats can adjust sensory acquisition based on internal cues

**DOI:** 10.1186/s12915-020-00904-2

**Published:** 2020-11-09

**Authors:** Arjan Boonman, Itai Rieger, Eran Amichai, Stefan Greif, Ofri Eitan, Aya Goldshtein, Yossi Yovel

**Affiliations:** 1 School of Zoology, Faculty of Life Sciences, Tel-Aviv University, 6997801 Tel Aviv, Israel; 2 Ecology, Evolution, Environment and Society Graduate Program, Dartmouth College, Hanover, NH 03755 USA; 3 Sagol School of Neuroscience, Tel-Aviv University, 6997801 Tel Aviv, Israel; 4 School of Mechanical Engineering, Faculty of Engineering, Tel-Aviv University, 6997801 Tel Aviv, Israel

**Keywords:** Sensory adjustment, Idiothetic cues, Doppler shift compensation, Echolocation, Active sensing

## Abstract

**Background:**

Sensory systems acquire both external and internal information to guide behavior. Adjustments based on external input are much better documented and understood than internal-based sensory adaptations. When external input is not available, idiothetic—internal—cues become crucial for guiding behavior. Here, we take advantage of the rapid sensory adjustments exhibited by bats in order to study how animals rely on internal cues in the absence of external input. Constant frequency echolocating bats are renowned for their Doppler shift compensation response used to adjust their emission frequency in order to optimize sensing. Previous studies documented the importance of external echoes for this response.

**Results:**

We show that the Doppler compensation system works even without external feedback. Bats experiencing accelerations in an echo-free environment exhibited an intact compensation response. Moreover, using on-board GPS tags on free-flying bats in the wild, we demonstrate that the ability to perform Doppler shift compensation response based on internal cues might be essential in real-life when echo feedback is not available.

**Conclusions:**

We thus show an ecological need for using internal cues as well as an ability to do so. Our results illustrate the robustness of one particular sensory behavior; however, we suggest this ability to rely on different streams of information (i.e., internal or external) is probably relevant for many sensory behaviors.

## Background

Sensory systems must constantly adjust according to the information they receive to improve sensory acquisition. How they do so have mostly been studied in the context of external input. Some examples include the following: pupils dilate and contract in response to changing illumination [1], the middle ear muscles contract to reduce the received level of loud noises [2], and the olfactory system habituates to long lasting odors [3]. Doppler shift compensation in bats is a prime example of a sensory adjustment [4]. Bats that use constant frequency echolocation signals (henceforth CF bats) adjust their emission frequency according to their speed in order to maintain the frequency of the returning echoes at the most sensitive range of their auditory system [5–9]. Because of the extremely narrow tuning of their hearing system [10], without Doppler shift compensation, CF bats would have real difficulties to detect the Doppler shifts created by insects’ wing beat, allowing them to detect prey. Moreover, the detection threshold of any object (including large obstacles) would be much higher. All past studies of this response showed that it is based on an external acoustic feedback, namely the bats assess the frequency of returning echoes and adjust their emission in order to ensure that the echoes are in the right frequency range. Supporting the notion that Doppler compensation is based on external feedback, bats that were flown in a wind-tunnel showed no compensation even though they were flapping vigorously in flight, while maintaining a ground speed of 0 m/s, thus not invoking a change in external feedback [6]. Moreover, it was reported in *Rhinolophus ferrumequinum* that if the temporal overlap between signal emission and echo reception is smaller than about 50%, bats will not Doppler compensate. This suggests that these bats compare the frequencies of the emitted signal and the received echo, thus depending on echoic feedback for proper sensing [11].

When external input is not available, idiothetic—*internal*—cues become crucial for guiding behavior. Internal sensory cues such as the vestibulo-ocular reflex [12] are well-known for their role in guiding movement, including in bats [13]. Can such internal cues also guide sensing and thus lead to sensory adjustments? In echolocation-based navigation and foraging, sensory feedback is not always available as bats sometimes fly in situations where they receive no echoes. This could happen for example, when a bat flies over a large gap in the canopy or when flying high above ground. In such situations, if the bat changes its speed and does not compensate for the new resulting Doppler shift, it might miss obstacle or target echoes. We thus hypothesized that bats should be able to perform it even without external echo feedback, by relying on internal cues.

The most common way to study Doppler compensation behavior is the pendulum paradigm. In this experiment, bats are restrained in a pendulum that is swinging towards a reflector while their emission frequency is measured as a function of their speed (we will refer to this condition as the “Feedback” condition [14–17]). The swinging of the pendulum is reminiscent of the foraging behavior of many CF bats that typically take off from a perch when prey is detected, intercept it, and land to feed. In our study, we used this same experimental paradigm, but our bats were swinging with no reflector anywhere around them, and thus, they could not use echo feedback to assess their speed (the “No Feedback” condition). We moreover used GPS tracking to examine whether bats sometimes fly too high to receive echoes.

## Results

**Fig. 1:**
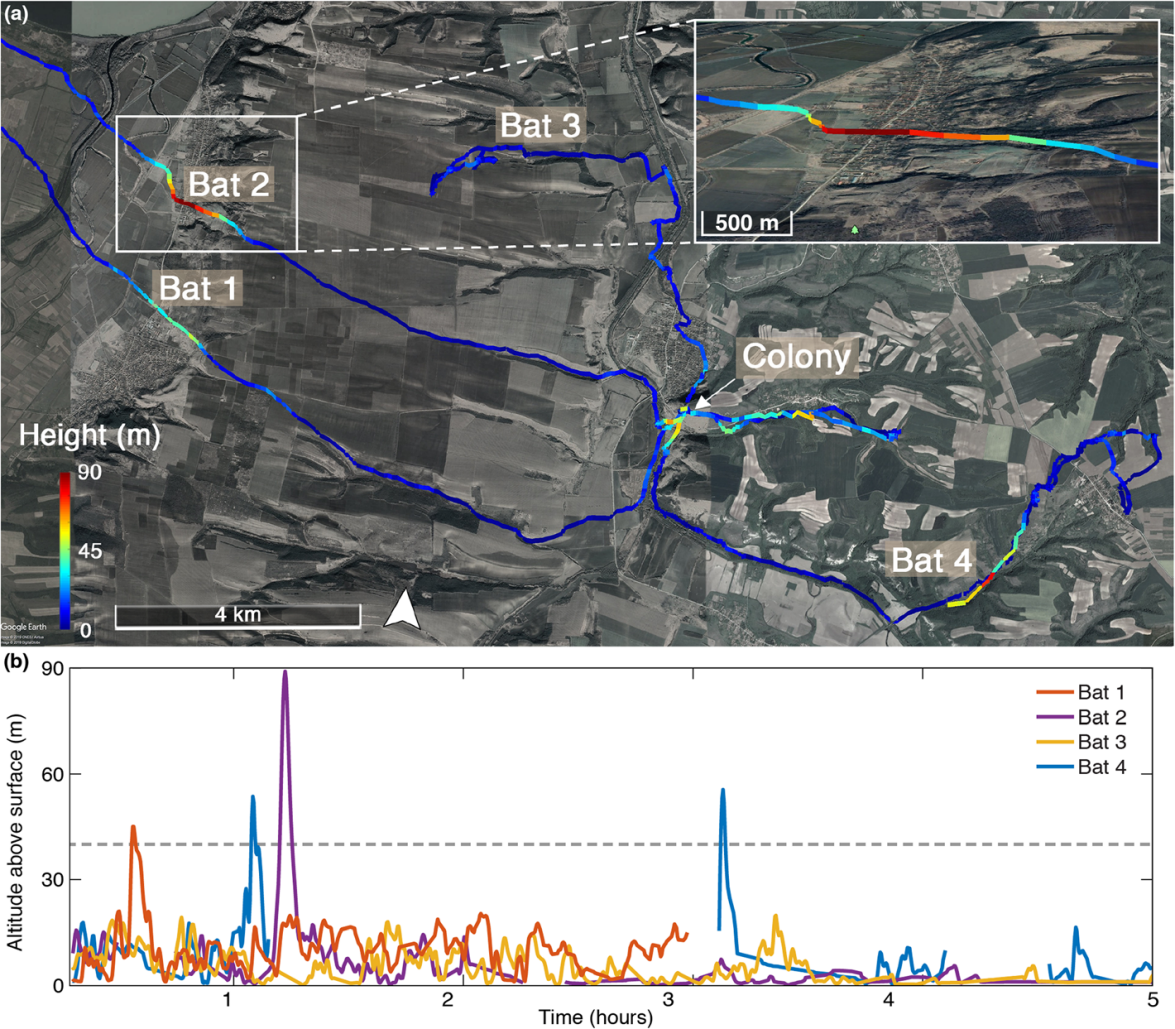
Bats fly without feedback. **a** Flight trajectory of four *R*. *ferrumequinum*. Flight altitude (above ground) is color coded on the map. Insert shows a zoom in on the ridge crossed by two of the bats. **b** Flight altitude of the four bats over time. Three of the four bats flew above 40 m for a few minutes at some point during the night, mainly when flying over a ridge that turns into a deep valley (see map in **a** to examine these occasions)

GPS data of three of the four *R*. *ferrumequinum* bats that we tracked revealed that they occasionally fly too high to receive background echoes. This happened, for example, at times when ground elevation changes rapidly. When crossing a ridge, three of the four bats flew for a few minutes (117 ± 69 s,) more than 40 m above ground, which should disable any echo reception (Fig. 1a, b). This finding shows that *R*. *ferrumequinum* bats find themselves deprived of echoes almost on a nightly basis.

**Fig. 2:**
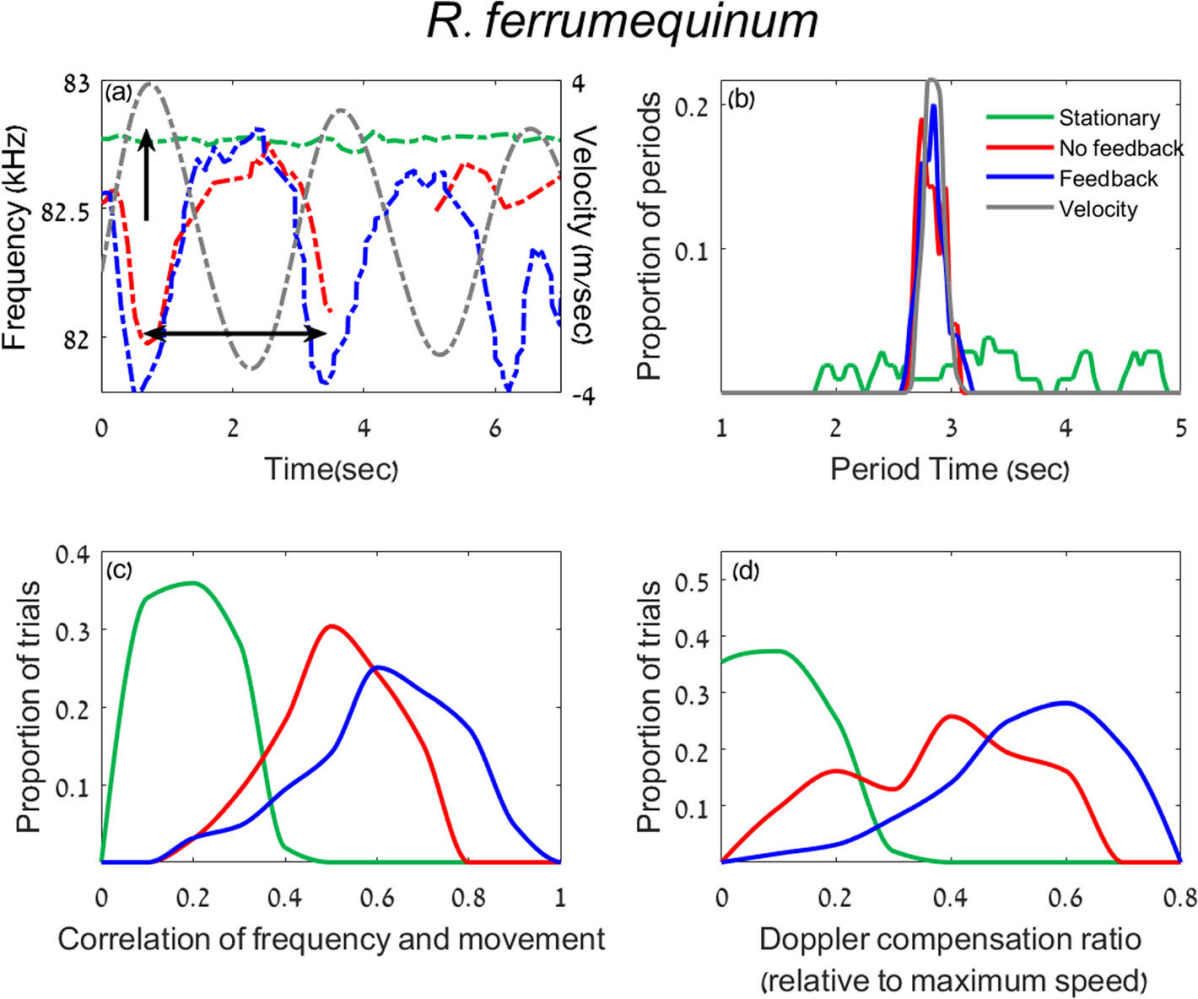
Doppler compensation without feedback in *R*. *ferrumequinum*. **a** An example of one trial showing the emission frequency with feedback (blue), the emission frequency without feedback (red), and the emission frequency of a Stationary trial (green). These colors are the same for all panels. The gray dashed curve shows the pendulum’s speed where positive speed means forward movement. A vertical arrow shows the point of maximum velocity when Doppler compensation should be maximal. A double-headed horizontal arrow shows one period of the movement and response. The response between 3.5 and 5 s without feedback (red) is absent because the bat did not call. All values in **b**–**d** are normalized between 0 and 1 to allow a comparison of the different units. Note that *ferrumequinum* frequencies in Israel are slightly higher than in Bulgaria. **b** The distribution of the period for the three conditions: Feedback, No Feedback, and Stationary. **c** The distribution of cross-correlation peak between the movement and the Doppler response for the three conditions. The cross-correlation was bound between 0 and 1 in our case with a perfect correlation represented by a value of 1. **d** The magnitude of compensation presented as a proportion of full compensation

**Fig. 3:**
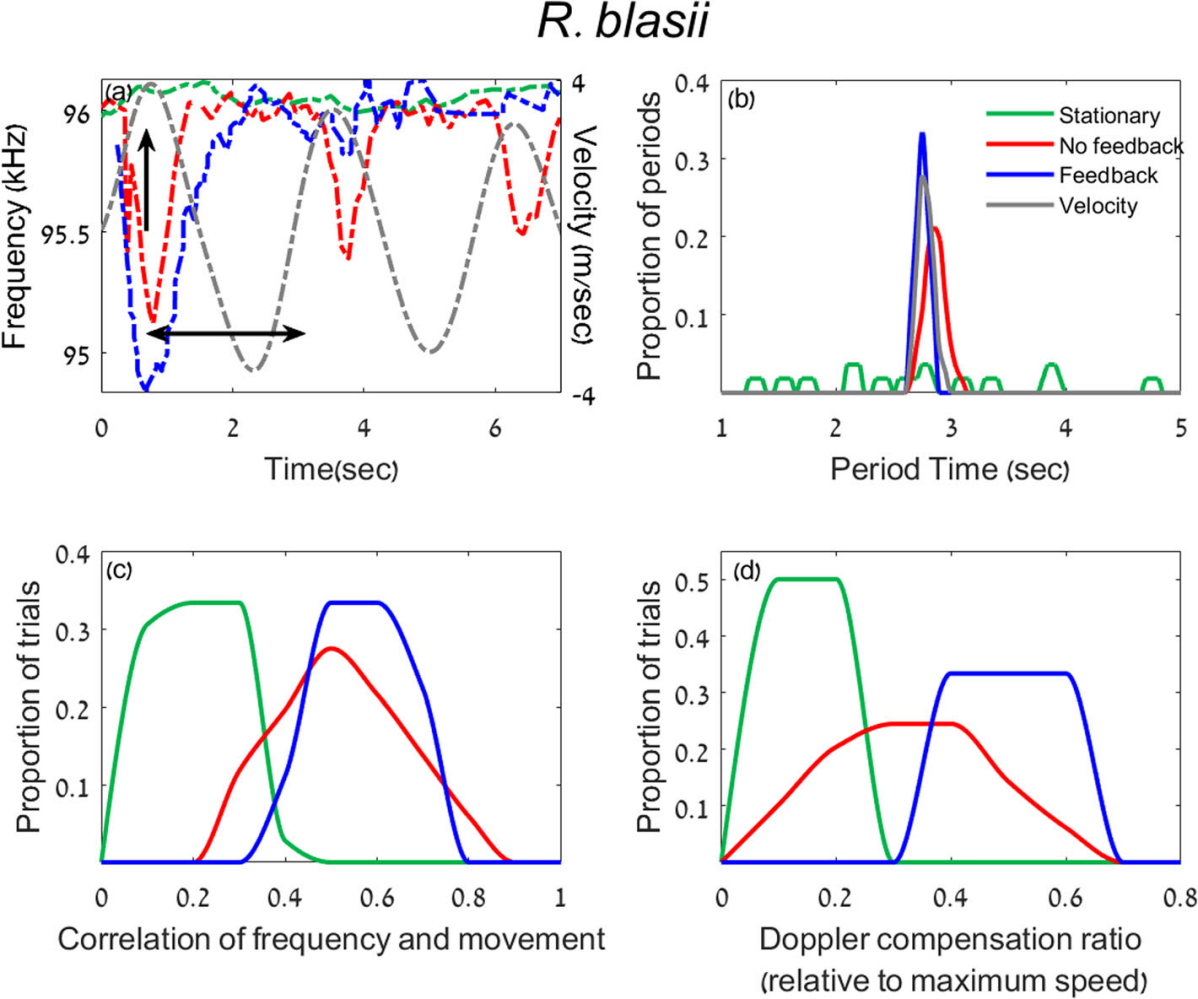
Doppler compensation without feedback in *R*. *blasii*. **a** An example of one trial. **b** The distribution of the period for the three conditions. **c** The distribution of cross-correlation peak between the movement and the Doppler response for the three conditions. **d** The magnitude of compensation presented as a proportion of full compensation. For more details, see Fig. 2 caption

To examine whether bats can indeed perform Doppler shift compensation without echoic feedback in a controlled experiment, we examined three species from the *Rhinolophidae* family using the pendulum paradigm (*R*. *ferrumequinum*, *R*. *blasii*, and *R*. *hipposideros*). In addition to the “No Feedback” and the “Feedback” conditions, we also recorded the bats while they were restrained in a stationary pendulum (without swinging) with no reflector in front of them (the “Stationary” condition). Bats of all species responded to the swinging by Doppler shifting their emission frequency both with and without feedback (Figs. 2a, 3a 4a). We used three different measurements in order to quantify the bats’ emission frequency which we will term the “Doppler response.” The first two aimed to examine the temporal accuracy of the response while the third analysis aimed to examine its spectral accuracy: (1) we compared the periods of the pendulum movement and the Doppler response, (2) we examined the cross-correlation between the Pendulum’s velocity and the Doppler response, and (3) we examined the magnitude of the Doppler shift in comparison with the expected one. See Additional file 1: Table S1 and Methods for the number of bats that responded in each treatment.

**Fig. 4:**
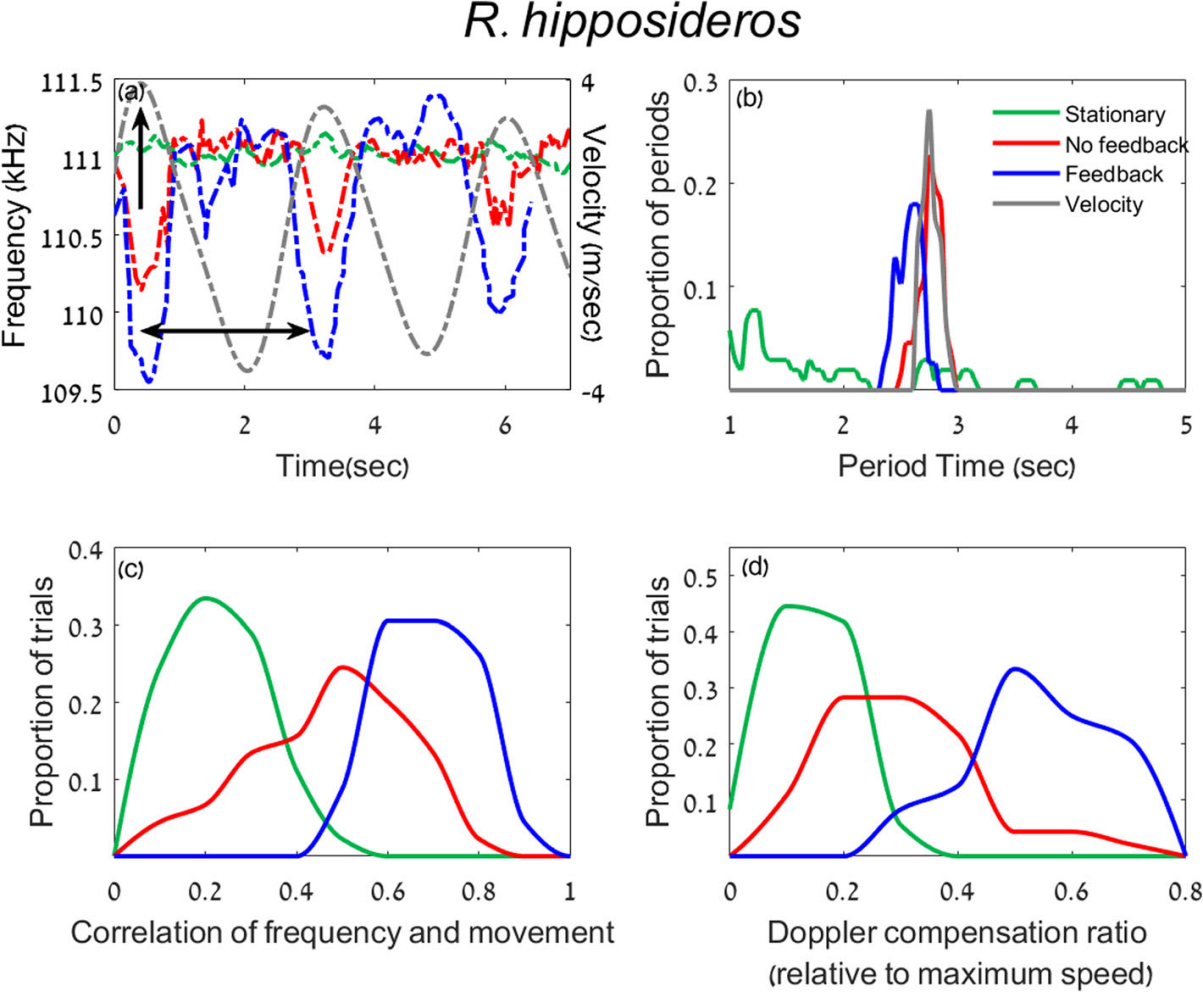
Doppler compensation without feedback in *R*. *hipposideros*. **a** An example of one trial. **b** The distribution of the period for the three conditions. **c** The distribution of cross-correlation peak between the movement and the Doppler response for the three conditions. **d** The magnitude of compensation presented as a proportion of full compensation. For more details, see Fig. 2 caption

1. To quantify the response and compare the Feedback vs. No-Feedback and the Stationary vs. moving conditions, we first estimated the period of the response. The pendulum’s oscillation period was 2.9 ± 0.3 s, and thus we hypothesized that if the bats responded to the movement, their response would be periodic and the period of their frequency shift should be similar to the period of the pendulum. We estimated the period of the bats’ frequency response by plotting the emission frequency over time and measuring the mean time difference between each two troughs (see horizontal arrow in Figs. 2a, 3a, 4a; the same criteria were used in all conditions, see the “Methods” section). When swinging *without feedback*, bats of all species shifted their emission frequency in a periodic fashion that was very similar to the pendulum’s periodic movement (Figs. 2, 3, 4 a and b; the mean periods were as follows (*n* represents number of trials): *R*. *ferrumequinum*—2.8 ± 0.1 *s* (*n = 11*); *R*. *blasii*—2.7 ± 0.1 *s* (*n = 17*); *R*. *hipposideros*—2.8 ± 0.1 *s* (*n = 16*)). In all species, these periods were significantly more similar to 2.9 s than expected by chance based on a permutation test on shuffled data (*P* = 0.01, *P* = 0.03, and *P* = 0.004 for the three *Rhinolophids* respectively, see the “Methods” section). In contrast, in the Stationary condition (when the pendulum was not moving), the response was not periodic and did not significantly differ from a random frequency shift (*P* = 0.3 (*n = 19*), *P* = 0.4 (*n = 12*), and *P* = 0.4 (*n = 16*) respectively, permutation test). As described many times before, the response in the Feedback condition (i.e., with a reflector in front of them) also showed a period that was significantly similar to the pendulum’s movement period (2.8 ± 0.1 *s* (*n = 23*), 2.8 ± 0.05 *s* (*n = 3*), 2.6 ± 0.1 *s* (*n = 8*) *P* = 0.004, 0.01, and 0.04 for the three species respectively, permutation test). As previously reported [18], the bats never shifted their frequency when the pendulum was moving backwards, not even in the Feedback condition, but see also [4] for electronic playback experiments.
2. Whereas the period is a measurement of the average rate of change, it does not reflect the momentary change of emission frequency. Two series of emission frequencies (which we will refer to as the frequency pattern) can have the same period but differ greatly in details. For example, one pattern could have a sinusoidal modulation while the other could be rectangular. In order to assess the overall temporal similarity of the pendulum’s velocity and the emitted frequencies, we calculated the (peak of the) cross-correlation between the two (see the “Methods” section). The frequency pattern in the No Feedback condition was significantly correlated with the velocity, suggesting that bats were adjusting their emission frequency based on the movement of the pendulum (Figs. 2, 3, 4c, *P* = 0.003 (*n = 11*), *P* = 0.001 (*n = 17*), and *P* = 0.003 (*n = 16*) for the three species respectively; permutation test on shuffled data). To control that the cross-correlation, we observed is indeed in response to the pendulum’s movement (and not random) we also cross-correlated the frequency pattern in the Stationary condition with the pendulum’s movement in the exact same way (we used the average pendulum velocity, the “Methods” section). In this case, there was no significant cross-correlation between frequency and velocity (*P* = 0.3 (*n = 19*), *P* = 0.2 (*n = 12*), and *P* = 0.3 (*n = 16*); for the three species). As expected, the maximum frequency shift occurred near the point of maximum forward velocity (see vertical black arrow in Figs. 2, 3, 4 a). The mean cross-correlation was higher in all three species in the Feedback condition (the cross-correlation for the Feedback vs. No Feedback conditions was *R* = 0.63 ± 0.15 (*n = 23*) vs. *R* = 0.5 ± 0.09 (*n = 11*), Mean ± SD in *R*. *ferrumequinum*, *P* = 0.01; *R* = 0.57 ± 0.07 (*n = 3*) vs. *R* = 0.53 ± 0.11 (*n = 17*) in *R*. *blasii*, and *R* = 0.68 ± 0.09 (*n = 8*) vs. *R* = 0.46 ± 0.14 (*n = 17*), in *R*. *hipposideros*. GLM with the cross-correlation peak set as the explained parameter, the condition as a fixed parameter and the individual and trials as random factors. Due to the small sample size in the “Feedback condition,” we could only calculate *P* values in *R*. *ferrumequinum*).
3. After showing that the temporal accuracy of the Doppler shift compensation response is high even without feedback, we next aimed to assess the accuracy of its magnitude. The bats’ Doppler shift response should supposedly ensure that the returning echo frequency is adjusted for the auditory fovea where the bats hearing sensitivity is best [19, 20]. Previous studies [9, 21] sometimes measured the accuracy of the Doppler shift response by comparing it to the reference frequency (*F*_ref_) which is supposedly the bat’s desirable received frequency. In order to assess *F*_ref_, researchers often take the bat’s mean received frequency in flight, but this measurement assumes that bats manage to compensate correctly and thus we wanted to avoid it. We dispensed with this assumption by using a different measurement of accuracy—we measured the momentary shift in the emitted frequency and then calculated the ratio between this momentary shift and the shift that would have fully compensated for the bat’s speed (i.e., we divide the actual shift by the pendulum-induced maximal Doppler shift). All three species shifted their frequency significantly more in the No Feedback condition than in the Stationary condition (Figs. 2, 3 and 4d; *P* = 0.02, *P* < 10^−5^, *P* < 10^−7^, GLM with the shift ratio as the explained factor and the rest as above, in *R*. *ferrumequinum*, *R*. *blasii*, and *R*. *hipposideros* respectively). On average, the bats always compensated more when feedback was available and this was significant for two species *R*. *ferrumequinum* and *R*. *hipposideros* (*P* = 0.003, *P* = 0.01, respectively, GLMs and above). The shift ratios in the Stationary, Feedback, and No Feedback conditions respectively were as follows: 0.07 ± 0.05 (*n = 19*), 0.54 ± 0.13 (*n = 23*), and 0.36 ± 0.18 (*n = 11*) in *R*. *ferrumequinum*, 0.09 ± 0.02 (*n = 12*), 0.47 ± 0.03 (*n = 3*), and 0.30 ± 0.12 (*n = 17*) in *R*. *blasii*, and 0.10 ± 0.04 (*n = 16*), 0.53 ± 0.07 (*n = 8*), and 0.29 ± 0.12 (*n = 16*) in *R*. *hipposideros* (Mean ± SD).

To further examine the magnitude of the compensation, we plotted the expected received echo frequencies in the bats’ ears, i.e., we summed the two-times Doppler effect due to the pendulum’s velocity with the bats’ emitted frequency. Analyzing the plots confirmed that by shifting their frequency, the bats manage to maintain a relatively constant received frequency with or without feedback (Additional file 2: Figure S1, there was no significant difference in the coefficient of variance (CV) of the frequency in *R*. *ferrumequinum* and *R*. *blasii P* > 0.05, GLM with the CV set as the explained variable, the condition as a fixed factor and the ID and trial as random effects in *R*. *hipposideros* the CV was actually significantly smaller without feedback, *P* < 10^−4^, but the effect size was small—0.002).

## Discussion

Our experiments demonstrate that bats can adjust their sensory acquisition based on idiothetic cues. We show that CF bats perform their well-known Doppler shift compensation behavior even when only relying on movement cues. The ability to respond without echoic feedback could be important for these bats when they are far from the ground as routinely happens in their everyday lives (Fig. 1). A bat that suddenly finds itself devoid of echo feedback could attempt to maintain a constant flight speed so that it does not need to adjust its emitted frequency. However, in case it decelerates (or accelerates), it will have to use internal cues in order to adjust its emitted frequency and maintain a potential received echo at its optimal (foveal) frequency. If it does not do so, it might miss-detect obstacles in its way. Note that it is very hard to maintain constant speed without sensory feedback and thus, this challenge is very probable. Acceleration could also occur during pursuit of prey or take off.

Bats could use different cues in order to Doppler compensate without echoic feedback. Two main hypotheses include (1) acceleration sensing by the vestibular system. Although integrating acceleration to velocity is probably a noisy process, there is evidence that mammalian brains can do so [22]. (2) Velocity sensing via somatosensory hair cells. Such sensors were described on bat wings [23–25]. Although in nature both mechanisms could assist bats, we hypothesize that in the pendulum experiments, bats were mostly relying on vestibular input because in our experiments, the bats were restrained in a device covering their body, so wing hair sensors would not be relevant for sensing velocity. Moreover, our bats were accurate in the timing of the response but less accurate in its magnitude. When Doppler shifting, bats must dynamically time their frequency shift, according to their momentary speed; and they must adjust the magnitude of the frequency shift such that it fully compensates for the Doppler shift. The magnitude of our bats’ response without feedback was smaller than with feedback—the frequency shift in the No Feedback condition was ca. ~ 55% smaller than when echoic feedback was available (Figs. 2, 3, 4c). This result is in line with a previous study that found that the magnitude of the Doppler shift compensation depends on echo-amplitude [26]. Temporally, on the other hand, the response without feedback (as assessed by the period and by the cross-correlation) was on a par with the response with feedback. This difference suggests use of the vestibular system. Estimating the timing of changes in speed is relatively easy when measuring acceleration, while estimating actual speed based on acceleration is more difficult. This difference might have also been intensified because of the (unnatural) movement of the pendulum. The swinging back and forth provides continuous acceleration cues, which can accurately drive temporal adjustments but make acceleration-based speed estimations difficult.

## Conclusions

In this study, we demonstrate how robust sensory acquisition can be, using external or internal information according to what is available. Future studies should look into how internal and external information are integrated when both are available. Examining such questions in human vision for example might be difficult because of the need to measure head and eye movements in a freely behaving subject. Measurable sensory responses such as the Doppler compensation thus provide unique opportunities to probe such questions.

## Methods

### Animals and experimental design

#### Pendulum experiments

All *Rhinolophus *species were captured and studied in Israel. Bats were captured under permit from the Israel Nature and Parks Authority (permit no. 2016/41422) who also approved the procedures. The researchers’ institutional ethics approval numbers are as follows: AB: TAU-R 1011346, EA: TAU-R 100493, and YY: TAU-R 100226 (other authors did not handle the bats). Bats were captured in two locations during emergence from their roost: *R*. *blasii* and *R*. *hipposideros* were captured using a mist net while emerging from a Cave in the Upper Galilee in northern Israel. All experiments were carried out in the field; see protocol below. *R*. *ferrumequinum* and *R*. *hipposideros* were captured using a harp-trap while emerging from a cave in the Samaria Hills near central Israel. In both sites, bats were processed upon capture (measured, sexed, and aged) and experiments were carried out in the order of capture. Bats were released upon completion of protocol (circa 15–20 min per individual) and the last bat was released before midnight, allowing for ample foraging time. In addition, 12 *R*. *ferrumequinum* were brought back to the lab for additional control experiments and released back into their cave the following evening. In total we tested the following number of bats: *R*. *ferrumequinum*—*15*, *R*. *blasii*—3, and *R*. *hipposideros*—4. The exact number of individuals that responded and were analyzed in the Stationary, No Feedback, and Feedback conditions respectively were 3,3,5 in *R*. *ferrumequinum* 3,3,1 in *R. blasii*, and 3,3,1 in *R*. *hipposideros* (see Additional file 1: Table S1).

#### On-board GPS recordings

Seven greater horseshoe bats (*Rhinolophus ferrumequinum*) were caught in North Bulgaria with a permit from the Bulgarian authorities MOEV-Sofia and RISOV-Ruse (permit number 189/07.08.2018). Bats (female, post-lactating) were caught in the late afternoon and weighed on average 23.5 g. We tagged them with a Vesper tag (ASD, inc.) that weighed 3.9 g, thus accounting for 16.5% of body weight on average (see [27] for details on tagging method). The GPS was set to record position every 15 s. The bats were released at the cave just after dark when the other bats were leaving the cave. The extra weight was high, but the tagging duration was very short, as 2 days later we managed to retrieve 5 tags (of which one had malfunctioned). We and others have shown that such short tagging periods have minor effects on bats [27–29].

#### GPS position analysis

Estimating altitude using GPS is noisy—the tag provides altitudinal data in meters above sea level (asl), and this data is then combined with topographical data to calculate altitude above ground for each recorded position. In a previous study, we estimated a SD of ~ 11 m [27]. However, three of the four bats ascended above 40 m (above ground), so even if we overestimated altitude by an extreme of 20 m, these bats should have had no access to ground echoes (see an estimation of *R*. *ferrumequinum* detection range below). Moreover, these ascent events always occurred when the bats crossed a point where ground elevation changed rapidly (due to flying over a ridge), so it is very reasonable that the bats changed their altitude above ground for short periods.

#### Pendulum experiments

We used a pendulum with an arm length of 2.1 m from the point of rotation to the point of the bat. The bat was fixed in a restrainer directed towards the direction of the forward swing and a 1/8″ microphone (Gras 40DP) was fixed just above it continuously recording the bat’s echolocation signals. The pendulum was released when the bat-restrainer was at an angle of ~ 50° above the horizon, leading to a maximum speed of 3.9 m/s at the bottom of its path. An IR video camera (Rogue Digital Video Camcorder or Sony HDR CX900E) was placed perpendicular to the plane of the pendulum’s movement and filmed the movement of the pendulum at a frame rate of 30 fps. Since the movement was planar to the camera, one camera sufficed to reconstruct the bat’s position. Motion reconstruction was done by means of a custom-written Matlab routine that converted inter-pixel distance into real distance units (calibrated based on arm’s length).

We subjected the bat to three different conditions: (1) motion in pendulum with no echo feedback—see below, (2) bat stationary with no echo feedback, and (3) motion in pendulum WITH echo feedback—with a wooden or brick wall in front of bat. To ensure that the bat could not use acoustic feedback, we carried out the experiments on an elevated platform suspended over a > 11-m vertical ground drop in the field experiments, and over the edge of the roof of a three-story building in the lab experiments, and we made sure that there were no reflective objects within range along the entire trajectory of the pendulum. To this end, for each species, we estimated the detection range of the bat and we made sure that there are no objects within this range (i.e., within a hemisphere of 180° around the bat) at each position along the trajectory. The detection range of each species was estimated assuming a 120-dB SPL emission level (at 10 cm) [30] and a 10-dB hearing threshold, while taking into account the atmospheric attenuation (at 25°, 70% humidity and sea-level air pressure), a 0-dB target strength (100% reflection), and the 2-way 6-dB spreading losses (assuming a wall, that is, maximum reflection). The bats’ maximum detection range would be < 10 m for *Rhinolophus ferrumequinum* (78 kHz; ~ 3.3 dB/m, emission frequency and atmospheric attenuation) down to < 6.9 m for *Rhinolophus hipposideros* (110 kHz; ~ 4.7 dB/m) and in between for *Rhinolophus blasii*. In all sites, the distance between the pendulum and the nearest object (including ground) was farther than these distances which are the maximal detection ranges. Note that these are maximal detection ranges—when the bat emits at full power and points its beam directly towards the nearest target (i.e., straight down) which is hardly ever the case. Moreover, for *R*. *ferrumequinum*, the bat with the longest detection range, we ensonified the path created by the pendulum swing and validated that no echoes were available. To this end, we used a Scan Speak R2604/833000 loudspeaker connected to an Avisoft DA converter through a Marantz MM7055 660 W amplifier. We emitted a FM-chirp signal (90–15 kHz) with maximum energy at 17 kHz (124 dB SPL at 10 cm from the speaker). We used an Avisoft CM16 microphone with the pre-amplifier at maximum gain, resulting in a 30-dB SPL noise floor. Only frequencies between 15 and 34 kHz gave rise to a detectable echo with the echo at 17 kHz being 68 dB weaker than the emission and at 34 kHz 90 dB weaker than the emission. Note that unlike the theoretical measurement above, this recording takes into account the actual reflectivity of the environment where the experiments took place. We could now extrapolate our results to the dynamic range of a *R*. *ferrumequinum*. To this end, *R*. *ferrumequinum* emits stronger signals than our speaker, but the attenuation at 80 kHz is at least 3 dB/m stronger than at 17 kHz. This means that the bat would need a dynamic range of ~ 150 dB in order to sense the nearest echo—a range far too large for any bat. The other species would need an even larger dynamic range as their emission frequencies are higher.

#### Excluding use of vision

Experiments were carried out at night at locations where the nearest visible light was at least 500 m away from the pendulum. During the swing the bat changed its height by up to 1.5 m thus accounting for an angular movement of the visual light of < 1° on the bat’s retina. All assessments of the visual acuity of Rhinolophid bats suggest a lower acuity (of 1–2° [13];) so the bats should not have been able to see the movement at all or perhaps see it with very poor resolution. In addition, for most bats, we directed a strong flash light towards their eyes from a short distance right before the swinging, to momentarily blind them.

#### Data acquisition and analysis

Emitted sounds were recorded “on board” with the Gras 40DP 1/8″ microphone connected to an Avisoft Hm116 amplifier sampling the signal at 375 kHz. We recorded audio during the first 3 cycles of the pendulum. At the swing-release, a clapping sound was produced to mark the release in the sound file. The sound files were analyzed with custom written scripts in Matlab. Each sound file was visually inspected, the clapping sound was manually detected, and the time of the emitted signals were calculated relative to it. Each emitted signal was automatically extracted and the frequency of its CF component was measured first with an FFT of 16,384 points, yielding a 22.9 Hz theoretical frequency resolution. Additional frequency measures were used to cross-validate the accuracy of the frequency measurements including the Chirp-Z transformation and the instantaneous frequency method (we examined the median, midpoint, and average of each signal). The correlation between the different methods was very high—*R*^2^ > 0.9 for all methods (Additional file 3: Table S2).

### Data pre-processing and parameter estimation

#### Exclusion criteria

We excluded trials that had less than 20 echolocation calls as well as trials that had no frequency shift (i.e., trials with shifts of less than 20% of the expected shift based on the pendulum’s maximum speed—3.9 m/s). Importantly, excluding trials where the bats did not show a clear shift in frequencies is legitimate because in this study, we ask whether bats can use internal cues to compensate for expected Doppler shifts and not when they do or do not use Doppler shift compensation. Our analysis aims to reveal whether any observed frequency shifts corresponded to the pendulum’s movement. We therefore removed trials in which no response was observed—this was done in all experimental conditions and we did not examine if the bats are more probable to show a response under different conditions (e.g., with or without feedback). No response behavior meant that the bat refused to call or called so sporadically that no meaningful analysis was possible. Many of the bats did not respond, but this is not surprising—there are many reasons for a bat, restrained in a very artificial situation (a pendulum swinging back and forth) to not perform Doppler shift compensation. In fact, also with feedback (when swinging in front of the wall), many of the bats did not respond. For example, in *R*. *ferrumequinum*, 20% of the bats had trials that followed the criteria above in the No Feedback condition and 30% did so in the Feedback condition.

Below, we describe how each of the specific analyses was performed. In all of the analyses, the same analysis was performed on the data in all conditions. We used three estimates to compare the bats, “Doppler response” with and without feedback: (1) we compared the periods of the pendulum movement and the Doppler response, that is, the time interval between two cycles of the response; (2) we examined the cross-correlation between the pendulum’s velocity and the Doppler response (i.e., between the velocity and the frequency vectors); and (3) we examined the magnitude of the Doppler shift in comparison with the expected one, i.e., how much did the bat shift its emission frequency. See Additional file 1: Table S1 and Methods for the number of bats that responded in each treatment.

#### Estimating the period

Before estimating the period, the emission frequency time series were smoothed with a moving average window of ~ 1.5 s. The peaks of the frequency series were found using the Matlab function findpeaks with setting the MinPeakDistance parameter to ca. 1.5 s. This same procedure was run on the stationary data to examine that it could not be explained by chance.

#### Calculating the cross-correlation

Before calculating the cross-correlation, the emission frequency time series were interpolated using a linear interpolation to a temporal resolution of 30 Hz (the same as the video recordings). Because none of the bats responded to the Doppler shift when the pendulum was moving backwards, for the cross-correlation analysis, we half-wave rectified the pendulum’s backwards velocity, leaving only the forward velocity (Additional file 4: Figure S2). Next, we normalized the frequency pattern to a range between 0 and 0.5 (and not between 0 and 1 because of the half-wave rectification). To estimate the cross-correlation, we also computed the auto-correlation of the velocity vector. We then divided the peak-to-peak maximum of the cross-correlation by the peak-to-peak maximum of the auto-correlation vector (see Eq. 1). A value of 1 would imply perfect correlation.

1 $$ \mathrm{Correlation}\ \mathrm{strength}=\frac{\mathrm{peak}-\mathrm{to}-\mathrm{peak}\ \mathrm{cross}\ \mathrm{correlation}\ }{\mathrm{peak}-\mathrm{to}-\mathrm{peak}\ \mathrm{auto}\ \mathrm{correlation}} $$

Because the movement of the pendulum was highly repetitive, we used the movement of a single representative trial per species. We used this same representative trial also when calculating the cross-correlation in the Stationary condition (when the pendulum was not moving). This control aimed to examine that an observed correlation in the moving condition could not be explained by some inherent behavior of the bats (which is not related to the movement).

#### Estimating the Doppler compensation magnitude

To estimate the Doppler compensation in each trial, we calculated the ratio between the maximum frequency shift and the maximum velocity induced Doppler shift. To calculate the maximum frequency shift, we subtracted the mean of the lowest 10% frequency measurements from the highest 10% measurements (we did not use the single minima and maxima to reduce the effect of outliers). This maximum frequency shift was then divided by the expected maximum Doppler shift which was calculated based on the maximum velocity according to Eq. 2:

2 $$ \varDelta f=\frac{2\bullet v}{c}{f}_{0.} $$

where $c\approx 343\ \left[\frac{m}{s}\right]$ is the speed of sound, *v* is the maximum speed of the pendulum estimated to be at $v\approx 3.9\ \left[\frac{m}{s}\right]$ multiplied by a factor of two because the bat experienced Doppler twice, and *f*_0_ is the resting frequency of each bat, taken as the mean of the highest 10% frequency values of the trial.

### Statistics

#### Testing the significance of the period and the cross-correlation

To test whether the period and the correlation could be explained by a random behavior, we shuffled the data. To this end, each frequency response (e.g., for bat X in trial Z of treatment Y) was shuffled multiple times until we had 1000 shuffled trials per species and condition for which we could estimate the distribution of the parameter in stake (i.e., period or cross-correlation). To quantify significance, we then quantified how many of the shuffled-based permutations had values higher than the mean real value or the two parameters (period and cross-correlation) for each species and each condition separately.

We used mixed model GLMs to compare different conditions (e.g., Feedback and No Feedback). We specify the definitions of the GLMS in the main text.

## Supplementary Information

**Additional file 1: Table S1.** The number of individuals who responded and the number of their trials which were analyzed in each condition.

**Additional file 2: Figure S1.** The received frequency. The expected received echo frequencies according to the bats’ emitted frequency and the momentary pendulum movement. We only show data for individual bats (of two species) for which we had data for both Feedback and No Feedback conditions. The pendulum’s velocity (pattern) is shown for comparison.

**Additional file 3: Table S2.** The correlation of the Chirp-Z frequency estimations with three alternative algorithms for five trials of swinging in front of wall (R^2^ values are presented).

**Additional file 4: Figure S2.** Estimating the cross-correlation. Left – The veolicty of the pendulum was normalized and half-reftified before cross-correlating it with the emitted frequency sequence. Right – The maximal peak-to-peak of the cross correlation function was divided by the maximum of the auto-correlation in order to assess the cross-correlation.

## Data Availability

All datasets and code generated during this study are available at Mendeley Data (DOI: 10.17632/6mtcs5p56m.2). Raw data are too large for archiving bur are saved on servers in TAU and are available upon request.
