# Supplementary material for: Echolocating bats can adjust sensory acquisition based on internal cues
Source: BMC Biol. 2020 Nov 9;18:166. doi: 10.1186/s12915-020-00904-2 (PMC7654590; doi:10.1186/s12915-020-00904-2)
Supplement: Supplementary file 1 — Additional file 1: Table S1. The number of individuals who responded and the number of their trials which were analyzed in each condition. [file 12915_2020_904_MOESM1_ESM.pdf]

**Species**	**ID**	**Condition**	**Number of recorded trials**	**Number of trials used**
ferrumequinum	1	stationary	7	6
ferrumequinum	9	stationary	6	6
ferrumequinum	D	stationary	9	7
ferrumequinum	1	no feedback	5	3
ferrumequinum	Nisrael2	no feedback	8	3
ferrumequinum	D	no feedback	5	5
ferrumequinum	1	feedback	5	5
ferrumequinum	2	feedback	5	3
ferrumequinum	9	feedback	5	5
ferrumequinum	D	feedback	6	6
ferrumequinum	F	feedback	6	4
blasii	1	stationary	9	6
blasii	2	stationary	6	3
blasii	C	stationary	7	3
blasii	1	no feedback	14	9
blasii	2	no feedback	12	2
blasii	C	no feedback	6	6
blasii	C	feedback	5	3
hipposideros	1	stationary	3	1
hipposideros	2	stationary	10	10
hipposideros	B	stationary	7	5
hipposideros	1	no feedback	7	7
hipposideros	2	no feedback	6	6
hipposideros	B	no feedback	5	3
hipposideros	B	feedback	8	8

Table S1. The number of individuals who responded and the number of their trials which were analyzed in each condition.
