# Supplementary material for: Echolocating bats can adjust sensory acquisition based on internal cues
Source: BMC Biol. 2020 Nov 9;18:166. doi: 10.1186/s12915-020-00904-2 (PMC7654590; doi:10.1186/s12915-020-00904-2)
Supplement: Supplementary file 2 — Additional file 2: Figure S1. The received frequency. The expected received echo frequencies according to the bats’ emitted frequency and the momentary pendulum movement. We only show data for individual bats (of two species) for which we had data for both Feedback and No Feedback conditions. The pendulum’s velocity (pattern) is shown for comparison. [file 12915_2020_904_MOESM2_ESM.pdf]

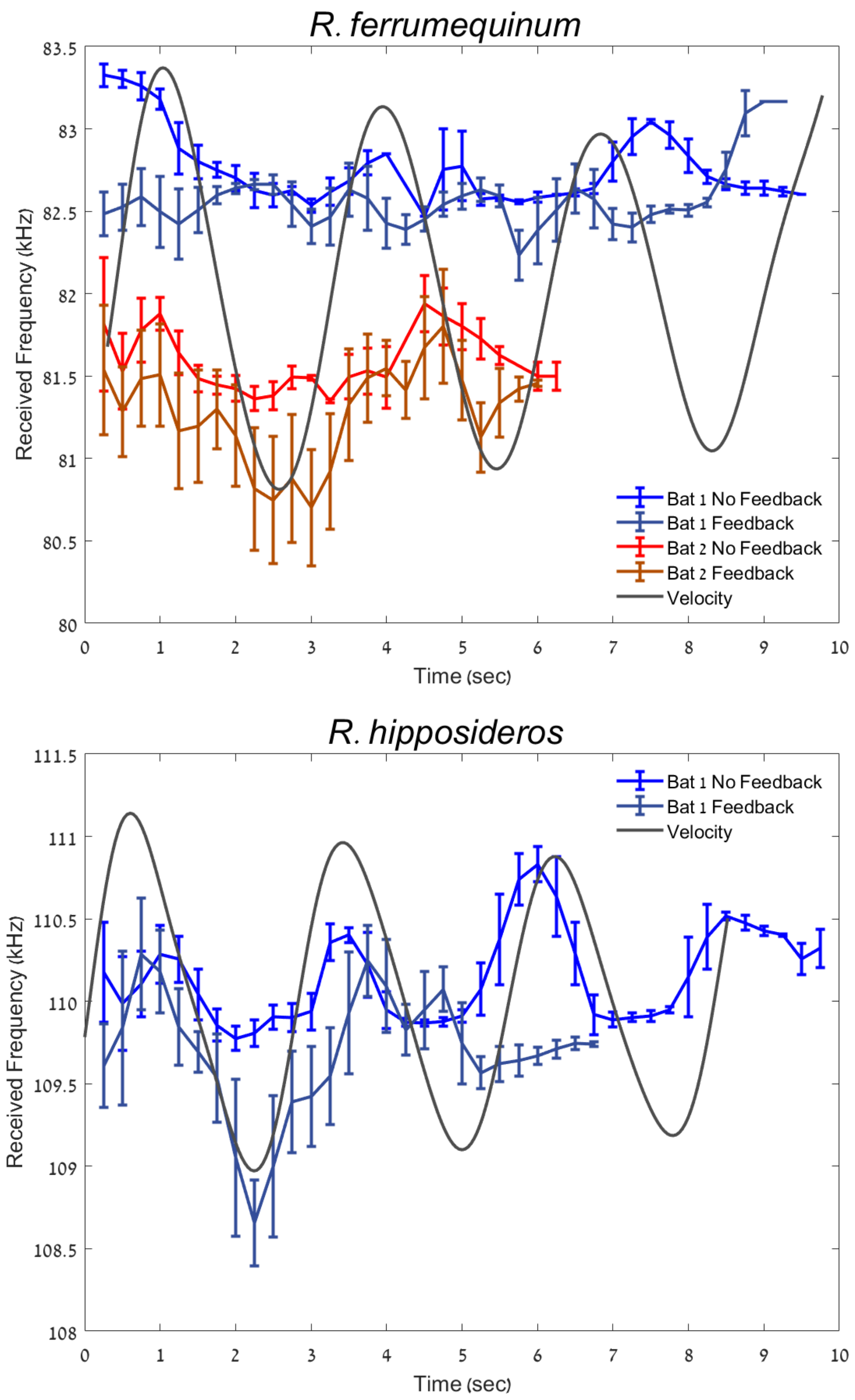

Figure S1. The received frequency. The expected received echo frequencies according to the bats' emitted frequency and the momentary pendulum movement. We only show data for individual bats (of two species) for which we had data for both Feedback and No Feedback conditions. The pendulum's velocity (pattern) is shown for comparison.
