# Supplementary material for: Echolocating bats can adjust sensory acquisition based on internal cues
Source: BMC Biol. 2020 Nov 9;18:166. doi: 10.1186/s12915-020-00904-2 (PMC7654590; doi:10.1186/s12915-020-00904-2)
Supplement: Supplementary file 3 — Additional file 3: Table S2. The correlation of the Chirp-Z frequency estimations with three alternative algorithms for five trials of swinging in front of wall (R2 values are presented). [file 12915_2020_904_MOESM3_ESM.pdf]

Trial	mid-Instant	median-Instant	Average frequency	FFT
1	0.97	0.99	0.98	0.99
2	0.92	0.98	0.96	0.99
3	0.91	0.98	0.98	0.99
4	0.95	0.99	0.98	0.99
5	0.95	0.99	0.99	0.999

Table S2. The correlation of the Chirp-Z frequency estimations with three alternative algorithms for five trials of swinging in front of wall (R^2 values are presented).
