# Supplementary material for: Echolocating bats can adjust sensory acquisition based on internal cues
Source: BMC Biol. 2020 Nov 9;18:166. doi: 10.1186/s12915-020-00904-2 (PMC7654590; doi:10.1186/s12915-020-00904-2)
Supplement: Supplementary file 4 — Additional file 4: Figure S2. Estimating the cross-correlation. Left – The veolicty of the pendulum was normalized and half-reftified before cross-correlating it with the emitted frequency sequence. Right – The maximal peak-to-peak of the cross correlation function was divided by the maximum of the auto-correlation in order to assess the cross-correlation. [file 12915_2020_904_MOESM4_ESM.pdf]

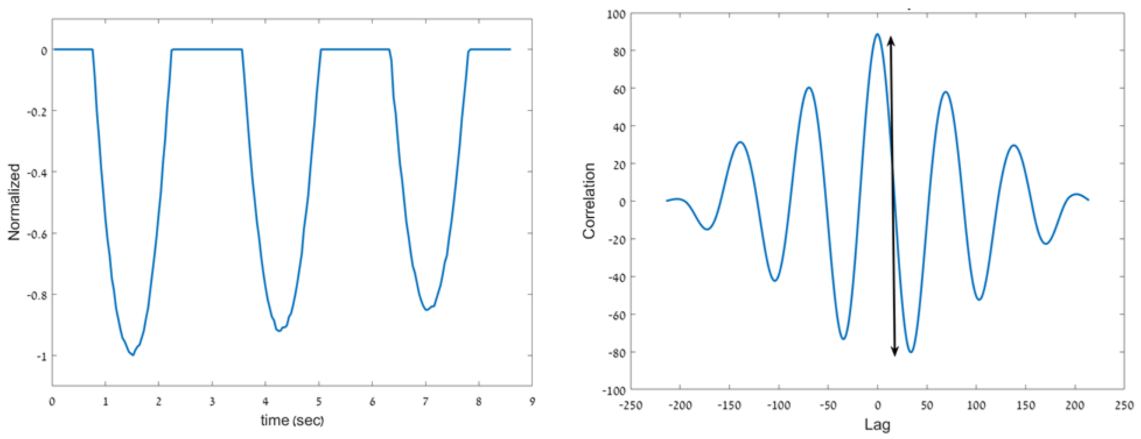

Figure S2. Estimating the cross-correlation. Left – The veolicty of the pendulum was normalized and half-reftified before cross-correlating it with the emitted frequency sequence. **Right –** The maximal peak-to-peak of the cross correlation function was divided by the maximum of the auto-correlation in order to assess the cross-correlation.
